# Neural mechanisms underlying social conformity in an ultimatum game

**DOI:** 10.3389/fnhum.2013.00896

**Published:** 2013-12-24

**Authors:** Zhenyu Wei, Zhiying Zhao, Yong Zheng

**Affiliations:** Key Laboratory of Cognition and Personality of Ministry of Education, School of Psychology, Southwest UniversityChongqing, China

**Keywords:** social conformity, behavioral change, ultimatum game, unfair treatment, fMRI

## Abstract

When individuals’ actions are incongruent with those of the group they belong to, they may change their initial behavior in order to conform to the group norm. This phenomenon is known as “social conformity.” In the present study, we used event-related functional magnetic resonance imaging (fMRI) to investigate brain activity in response to group opinion during an ultimatum game. Results showed that participants changed their choices when these choices conflicted with the normative opinion of the group they were members of, especially in conditions of unfair treatment. The fMRI data revealed that a conflict with group norms activated the brain regions involved in norm violations and behavioral adjustment. Furthermore, in the reject-unfair condition, we observed that a conflict with group norms activated the medial frontal gyrus. These findings contribute to recent research examining neural mechanisms involved in detecting violations of social norms, and provide information regarding the neural representation of conformity behavior in an economic game.

## INTRODUCTION

We live in a highly complex social environment where social information continuously affects perception and decision-making. Previous studies have shown that individuals systematically change their opinions and behaviors in order to align with group norms (Cialdini and Goldstein, 2004). This phenomenon is known as “social conformity,” and refers to the action of changing one’s initial choices or opinions to match those of the group majority (Turner, 1991).

Following the work of Asch (1951), psychologists have extensively examined the causes and underlying mechanisms of social conformity. There are three motivations related to conforming behavior: a desire to be correct, a desire to obtain social approval from others, and a desire to maintain a positive self-concept (Cialdini and Goldstein, 2004). Recent studies have focused on the neural basis of social conformity. Klucharev et al. (2009) found that conflict with group opinions triggered a neuronal response in the dorsal region of the posterior medial frontal cortex and the ventral striatum – brain areas that are often involved in reward processing and behavioral adjustments (Berns et al., 2001; Holroyd and Coles, 2002). Signal changes in these regions predicted subsequent adjustments of behavior in line with that of the group (Klucharev et al., 2009). A follow-up study indicated that transient down-regulation of the posterior medial frontal cortex by theta-burst transcranial magnetic stimulation (TMS) reduced conformity, suggesting that a fundamental performance-monitoring neural mechanism underlies social influence (Klucharev et al., 2011). Two fMRI studies also revealed that social information could change participants’ initial judgments and affect neural activity within relatively low-level processing areas associated with each task (Berns et al., 2005, 2010). Another study found that social influence was accompanied by modulated engagement of two brain regions linked to the coding of subjective value – the nucleus accumbens and orbitofrontal cortex; this result demonstrated that exposure to group opinions could affect individual neural representations of the subjective value assigned to stimuli (Zaki et al., 2011). Additionally, during a music choice task, activity in the ventral striatum – involved in object evaluation – suggested that social influence mediates the basic value signals in known reinforcement learning circuitry (Campbell-Meiklejohn et al., 2010).

By combining game theory models with psychological and neuroscientific methods, researchers have begun to investigate the psychological and neural correlates of social decision-making. This neuroeconomic approach can extend our knowledge of the brain mechanisms involved in social decisions (Sanfey, 2007). Decision researchers have focused on the fundamental impact of social factors on human behavior. Many of our decisions depend on the concomitant choices of others (Sanfey, 2007). According to the social influence hypothesis, humans are influenced in their beliefs and behaviors by the preferences and behaviors of others (for a review, see Haun and van Leeuwen, 2012; Morgan and Laland, 2012). Therefore, the present study assessed whether the choices of individuals during a monetary game could be modulated by peers’ opinions. We were also interested in the neural mechanisms underlying this phenomenon.

The ultimatum game (UG) is often used to examine responses to fairness (Güth et al., 1982). In the original UG, one player (the proposer) allocates money to himself/herself and to another player (the responder). The responder can either accept or reject the offer. If the responder accepts, both players win their respective amounts, but if the responder rejects, both players receive nothing. Results have shown that people reject a high proportion of unfair offers, which would not be adaptive from a rational perspective (Camerer, 2003). In the present study, we developed a variant of the UG in which participants were asked to decide whether to accept or reject an offer provided by a proposer. After the subject made his/her initial choice, he/she was informed of the choices from four other peers, which could be incongruent, moderately incongruent, or congruent with his/her choice. Then, the participant was given a second opportunity to decide whether to accept or reject the same offer.

We predicted that participants would change their choices once they found out that their decisions did not match those of the majority of the group. To examine the neural mechanisms related to social conformity during monetary allocation decision-making, we employed functional magnetic resonance imaging (fMRI). With the assumption that social influence affects our behavior through the mechanisms involved in behavioral adjustments (Klucharev et al., 2011), we hypothesized that a conflict with the group opinion would enhance activity in regions involved in norm violations and behavioral adjustment. These regions play a key role in conforming behavior. Further, we predicted that the initial choice type and offer type might affect brain responses to external information.

## MATERIALS AND METHODS

### PARTICIPANTS AND DESIGN

Thirty healthy right-handed participants (mean age = 21.6, female = 15) participated the experiment. These participants were recruited from Southwest University through advertisements. All were native Mandarin speakers, with no neurological illness as confirmed by psychiatric clinical assessment or psychological disorders, and with (corrected to) normal color vision. Written informed consent was obtained after detailed explanation of the experiment. The study was approved by the Ethics Committee of Southwest University.

Data from one participant was excluded from the study due to head movements exceeded 2.5 mm. This resulted in 29 participants for final analyses (14 males). The experiment used a two-factor within-participant design with two types of offer (fair offer and unfair offer) and four levels of group choice (incongruent, moderately incongruent, congruent, and no information).

### STIMULUS MATERIALS

Previous behavioral research indicated that the average proposer offers 40% of the amount to the responder, and 16% of the offers are rejected (Oosterbeek et al., 2003). Low offers, around 20% of the total sum, have about a 50% chance of being rejected (Güth et al., 1982; Bolton and Zwic, 1995; Nowak et al., 2000). There were 140 offers (70 fair offers and 70 unfair offers) in the present study. All fair offers split the money according to ratios ranging from 50:50 to 60:40, while unfair offers offered 10–20% of the total sum to the responder. The offer stimuli consisted of the sum of money and the distribution plan. The former was presented in the upper portion of the picture and the latter in the lower part of the picture. The number under “you” was assigned to the subject and the number under “proposer” was assigned to the proposer.

Participants’ peer choices were presented in the form of a table. Each was placed in the corresponding box. The number “1” indicated a choice to accept the offer, and the number “2” indicated a choice to reject it. There were four conditions of social influence: incongruent (the participant’s initial choice differed from the choices of three or four peers); moderately incongruent (the participant’s initial choice was inconsistent with the choices of two group members); congruent (the participant’s initial choice was consistent with the choices of three or four group members); and no information (the four numbers were replaced with “×”).

### EXPERIMENTAL PROCEDURES

When each participant arrived at the laboratory, she/he and four players were told that they would perform the experiment in separate rooms. In the experiment, they would play a monetary game independently against a human proposer, who would be in the MRI waiting room. They would see the choices of others through a local network on computers in the experiment. They were then asked to fill out a questionnaire together in the MRI waiting room. After that, the four players left the MRI waiting room accompanied by an assistant.

**FIGURE 1:**
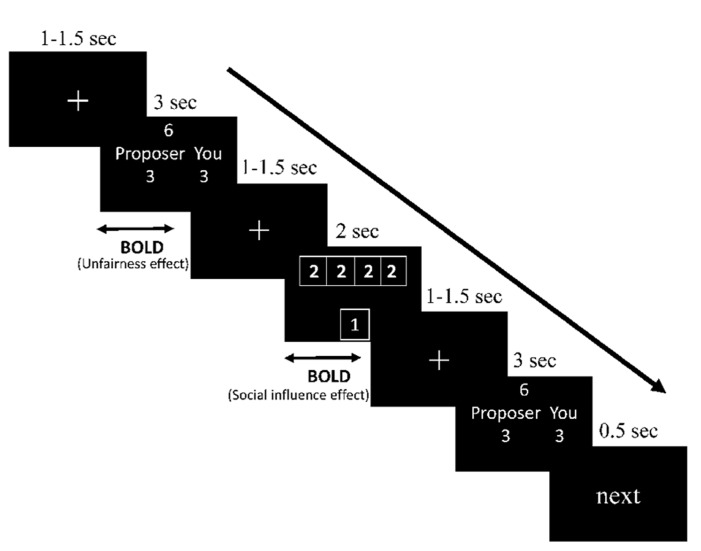
**Demonstration of sequence of events in a trial (take fair offer and incongruent information condition as an illustration)**.

Participants then received instructions about the procedure of the experiment. At the beginning of each trial, the participants were presented with a fixation point for a duration of 1–1.5 s. The offer proposed for all responders would be shown on the screen for 3 s, followed by another, identical, fixation point. Participant used the index and middle fingers of their right hand to respond to the offer by pressing one of the two buttons on a MRI-compatible button box (“1” to accept and “2” to reject the offer). The choices of others along with the participants’ then replaced the fixation point for 2 s, after which another variable interval lasting 1–1.5 s was presented. Finally, the same offer was presented for 3 s again, and the participant was asked to decide on it (to accept or to reject) again. The offer was followed by the word “next” being displayed for 500 ms, which indicated that the next trial was about to begin. The sequence of events in a trial is illustrated in **Figure 1**.

The experiment contained two blocks (70 trials each; 140 trials in total). On average, a trial lasted 13 s. In 20 of the trials, the participant’s initial choice was fixed to be inconsistent with the choices of two peers – in other words, consistent with the choices of two peers. These trials were not included in the final analysis because they were used solely to maintain believability of the interaction between participant and the four peers. In one third of the remaining trials (40 trials), the group’s choices were hidden from the subject (the no information, or baseline condition). For the 40 trials of the incongruent condition, three or four group members’ choices differed from the choice of the participants. For the 40 trials of the congruent condition, one or none of the group members’ responses was inconsistent with that of participants. Before performing the task in the scanner, participants completed a training session that used four different offers; during this task, the choices of the other group members remained hidden.

A PC running E-Prime 2.0 was used to display the stimuli and acquire the responses of the participants, as well as the reaction time (RT). Subjects viewed the experiment task by a mirror placed on the top of the image acquisition coil which could reflect the screen mounted at the back of the scanner.

### IMAGE ACQUISITION

Functional MRI data were acquired using a 3T Siemens Trio scanner. Each scan contains 435 functional volumes, using an echo-planar imaging (EPI) sequence with the following parameters: TR/TE = 2000/30 ms, flip angle = 90°, acquisition matrix = 64 × 64, FOV = 192 mm × 192 mm, axial slices = 32, thickness/gap = 3 mm/1 mm, voxel size = 3 mm × 3 mm × 3 mm. The first three images were discarded for the saturation effect.

### DATA ANALYSIS

Image preprocessing was performed with statistical parametric mapping 8 (SPM8; Welcome Department of Imaging Neuroscience, University of London, UK). Functional images were first corrected for motion artifacts. Then images were interpolated to correct for slice timing, and spatially normalized into the Montreal Neurological Institute (MNI)-space using the SPM8 EPI template, and resampled into 3 mm × 3 mm × 3 mm voxels. Images were smoothed using an 8 mm^3^ full-width-at-half-maximum (FWHM) Gaussian kernel.

Statistical analysis was performed in a general linear model in SPM8. The regressors were included based on offers (fair offers and unfair offers), external information (incongruent, congruent, and no information), and a combination of these factors. These regressors were then convolved with the standard hemodynamic response function. In addition, the realignment parameters were included to regress out potential movement artifacts.

For a whole-brain analysis, the results from random effects analyses were all initially thresholded at *p *< 0.001 (uncorrected) and further corrected using AlphaSim provided by REST (Song et al., 2011). For all AlphaSim corrections, cluster radius connection: rmm = 5, and 3d FWHM were estimated with their corresponding Residual images. 1000 iterations were performed using Monte Carlo method. Contrasts were generated from the design matrix at the individual level, which were then entered into a second-level group analyses. The effect of unfairness was estimated by a 2 (choice: accept, reject) × 2 (offer type: fair, unfair) ANOVA, the brain areas involved in the presentation of external information were generated by contrasting the incongruent effect (*incongruent condition* > *no information; incongruent condition* > *congruent condition*) and the congruent effect (*congruent condition* > *no information; congruent condition* > *incongruent condition*). For more details insights into which brain regions play a critical role in conforming behavior, we contrasted brain responses to incongruent group opinion followed by conformity with responses to incongruent group opinion followed by non-conformity. For explore whether the initial choice type and offer type can affect the brain responses to external information, we analyzed the interaction among the initial choice type (within group factor: accept versus reject), the offer type (within group factor: fair versus unfair), and the social influence (within group factor: incongruent versus congruent). Regions are projected onto a surface template (Caret software, Van Essen, 2005).

## RESULTS

### BEHAVIORAL RESULTS

Trials in which the subjects did not respond within decision time window in the first and/or second decision stage were excluded from further data analyses. There are 4.2% of total trials were rejected to enter the following data analyses.

#### Unfairness effect

A 2 (choice: accept, reject) × 2 (offer type: fair, unfair) ANOVA was used to analysis the RTs in the initial presentation of offers. The interaction between choice and offer type was significant, *F*(1,28) = 5.26, *p *< 0.05. The result indicated that responses were faster when subjects rejected the fair offers (*M *= 832.83, *SD *= 132.24) than when they rejected the unfair ones (*M *= 1158.84, *SD *= 88.86),* F*(1,28) = 4.545, *p *< 0.05. Regarding the participants’ choices, we found that subjects rejected the unfair offers (64%) at a significantly higher rate than the fair offers (16%), *t*(28) = -5.235, *p *< 0.001.

#### Social influence effect

Conformity effect was measured by the rate of change of participants. A 2 (initial choice: accept, reject) × 2 (offer type: fair, unfair) × 2 (external information: incongruent, congruent) ANOVA revealed a significant main effect of the factor *external information*, *F*(1,28) = 4.479, *p *< 0.05. Subjects changed their initial choices at a significantly higher rate in incongruent condition (*M *= 0.2, *SD *= 0.04) than in congruent condition (*M *= 0.11, *SD *= 0.03). The interaction among initial choice, offer type and external information was not significant. To test whether participants changed their initial choices after they rejected the unfair offers, we conducted a contrast between unfair-reject-incongruent and unfair-reject-congruent condition. The result showed that participants changed their initial choices at a significantly higher rate in incongruent condition (*M *= 0.15, *SD *= 0.3) than in congruent condition (*M *= 0.02, *SD *= 0.05), when they rejected the unfair offers in the first decision phase, *t*(28) = -2.39, * p *< 0.05.

### fMRI RESULTS

#### Unfairness effect

**FIGURE 2:**
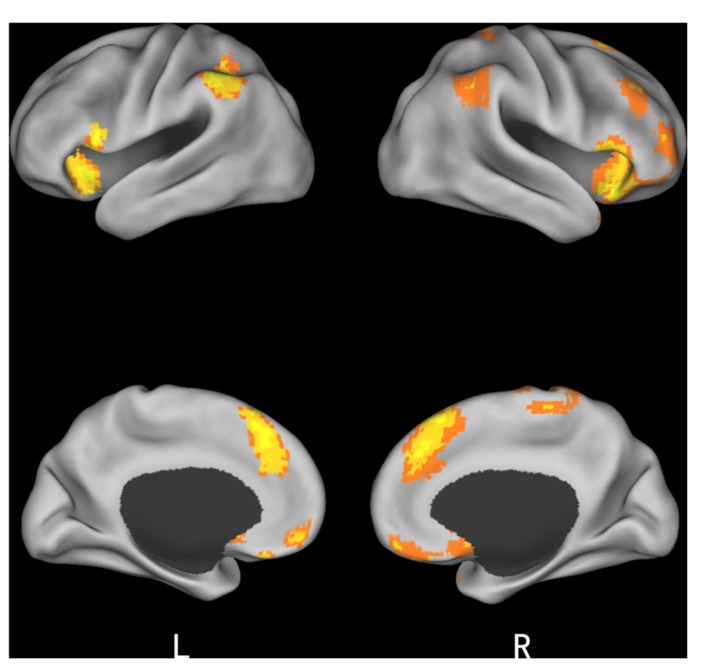
**Brain regions involved in the initial choice type and offer type interaction**.

To identify brain regions involved in the perception of unfair treatment in the first presentation of offers, we conducted a 2 (*initial choice*: accept, reject) × 2 (*offer* type: fair, unfair) ANOVA. The interaction was significant in several brain regions, including the bilateral insula, medial prefrontal cortex (mPFC), anterior cingulate cortex, precuneus, and bilateral inferior parietal lobule (IPL). *Post hoc* contrast indicated that these brain regions were activated when participants rejected the fair offers and accepted the unfair offers (see **Table 1** and **Figure 2**).

**Table 1:** Significant activation clusters for the interaction between choice and offer type.

Brain region	HEM	*x*	*y*	*z*	No. of voxels	*F*-value
Insula	R	33	15	-9	44	43.29
Insula	L	-48	9	12	47	32.95
mPFC	R	6	27	57	174	38.33
IPL	L	-54	-48	45	100	26.71
IPL	R	54	-54	-54	169	28.49
ACC	R	6	48	-12	34	23.03
Precuneus	R	9	-33	60	16	25.8

*
p < 0.005 (AlphaSim correction, cluster size > 242 voxels, FWHMx = 18.361, FWHMy = 16.701, FWHMz = 17.705). HEM, hemisphere; mPFC, medial prefrontal cortex; ACC, anterior cingulate cortex; IPL, inferior parietal lobule.*

#### Social influence effect

**FIGURE 3:**
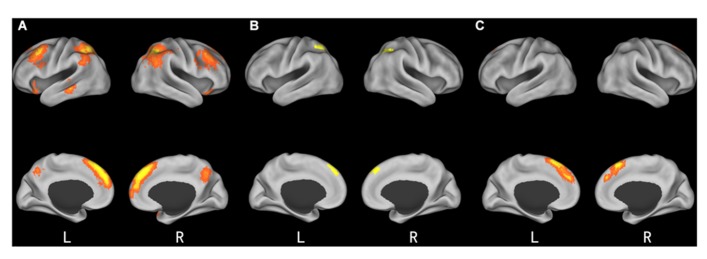
**Brain regions involved in social influence. (A)** incongruent > baseline; **(B)** congruent > baseline; **(C)** incongruent > congruent.

**FIGURE 4:**
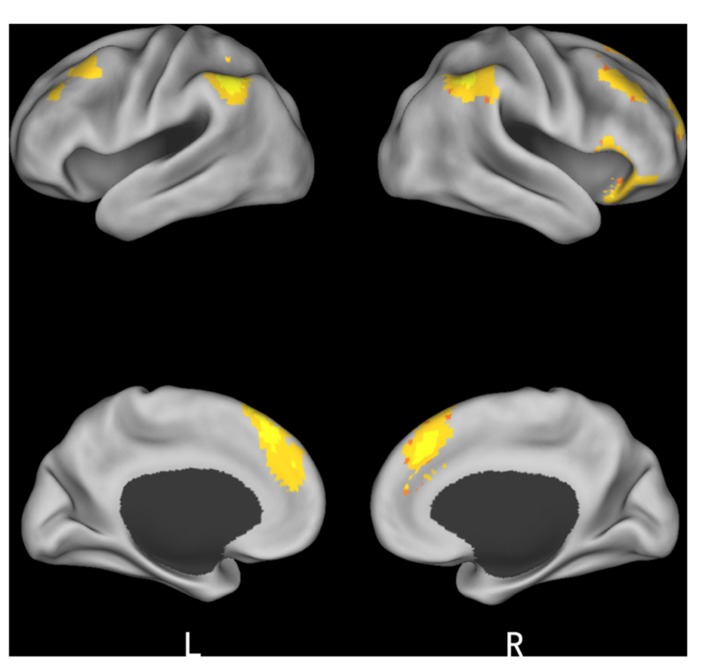
**Brain regions involved in conformity effect**.

As expected, the difference between incongruent condition and baseline condition (no information) induced activation in bilateral insula, middle temporal gyrus (MTG), bilateral middle frontal gyrus (MFG), bilateral IPL, mPFC, and precuneus. Comparison of activity in congruent condition with baseline condition showed significantly greater activation in bilateral superior parietal lobule (SPL) and superior frontal gyrus (SFG) when participants viewed congruent peers’ choice. And we also compared neural activity in the incongruent condition and congruent condition. Incongruent condition activated the (mPFC, see **Table 2** and **Figure 3** for details). Then, we compared the incongruent trials which subjects changed their initial choices with the trials which subjects didn’t change their initial choices. The result showed that the neural activity in the insula, bilateral MFG, medial prefrontal cortex (mPFC), bilateral IPL, and midbrain elicited by the social conflict following conformity were stronger than the activity elicited by the social conflict following non-conformity (see **Table 3** and **Figure 4** for details).

**Table 2:** Significant activation clusters for social influence.

Brain region	HEM	*x*	*y*	*z*	No. of voxels	*t*-value
***Incongruent* > *baseline***
Insula	R	30	18	-18	25	4.77
Insula	L	-30	18	-18	22	4.73
MTG	L	-63	-36	-9	49	4.19
MFG	R	48	21	42	130	5.31
MFG	L	-33	6	51	203	4.64
IPL	R	36	-54	39	176	6.26
IPL	L	-33	-51	48	140	5.91
Precuneus	R	3	-60	39	54	4.42
mPFC	L	-9	30	51	420	7.81
***Congruent* > *baseline***
SPL	L	-30	-66	57	48	4.5
SPL	R	36	-54	39	60	4.82
SFG	L	-3	42	42	34	4.89
***Incongruent* > *congruent***
mPFC	L	-6	21	57	98	6.17
***Congruent* > *incongruent***
No cluster

*p < 0.05 (AlphaSim correction, cluster size > 37 voxels, FWHMx = 11.918, FWHMy = 11.131, FWHMz = 12.28). HEM, hemisphere; MTG, middle temporal gyrus; MFG, middle frontal gyrus; IPL, inferior parietal lobule; mPFC, medial prefrontal cortex; SPL, superior parietal lobule; SFG, superior frontal gyrus.*

**Table 3:** Significant activation clusters for conformity effect.

Brain region	HEM	*x*	*y*	*z*	No. of voxels	*t*-value
***Changed* > *unchanged***
Insula	R	54	21	-6	45	5.89
MFG	R	30	57	15	25	4.27
MFG	R	42	33	42	42	5.66
MFG	L	-42	24	48	150	5.06
IPL	L	-48	-60	57	57	6.58
IPL	R	48	-48	42	300	6.03
Midbrain	L	-12	-9	0	14	4.8
mPFC	R	6	30	42	320	7.12

*p < 0.05 (AlphaSim correction, cluster size > 79 voxels, FWHMx = 15.533, FWHMy = 13.278, FWHMz = 13.553). HEM, hemisphere; MFG, middle frontal gyrus; IPL, inferior parietal lobule; mPFC, medial prefrontal cortex.*

**FIGURE 5:**
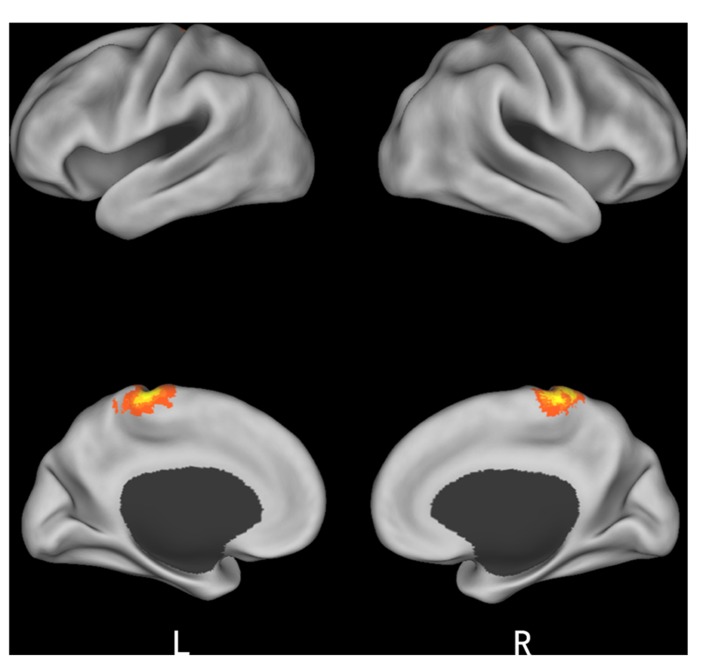
**Brain regions involved in the interaction of offer type and social influence when subjects viewing others’ choices after they rejected the offer in the first decision phase**.

Finally, we studied a 2 (*initial choice*: accept, reject) × 2 (*offer* type: fair, unfair) × 2 (*social influence*: incongruent, congruent) ANOVA. Only the effect of *social influence* × *offer type* interaction was significant in the medial frontal gyrus when subjects viewing others’ choices after they rejected the offers in the first decision. *Post hoc* contrast revealed that the neural activity in the medial frontal gyrus was higher for unfair-reject-incongruent condition than for fair-reject-incongruent condition, *p* < 0.001. (see **Table 4** and **Figure 5** for details).

**Table 4:** Significant activation clusters for the three-way ANOVA.

Brain region	HEM	*x*	*y*	*z*	No. of voxels	*F*-value
MFG	L	-3	-36	63	12	22.36

*p < 0.05 (AlphaSim correction, cluster size > 101 voxels, FWHMx = 17.599, FWHMy = 15.44, FWHMz = 15.622). HEM, hemisphere; MFG, medial frontal gyrus.*

## DISCUSSION

The present study suggests that individuals are likely to be influenced by others’ opinions and conform to the behavior of their peers. Our results showed that participants changed their initial choices in a monetary game when those choices differed from the majority choice of the group to which they belonged. We found that participants rejected a greater number of unfair offers. The rate of change was higher when participants initially rejected the unfair offer in the incongruent condition than in the congruent condition. These results help to explain previous findings showing that group decisions were very different from individual decisions: groups were more willing to accept unfair offers than were individuals during the UG (Bornstein and Yaniv, 1998). In the present study, individuals changed their decisions in response to unfair treatment due to peer pressure and a preference for conformity.

As previous research suggests, the neural mechanisms underlying social conformity are similar to the fundamental neural mechanisms involved in behavioral adjustments (Klucharev et al., 2009, 2011). Our functional imaging data suggest that being incongruent with group opinion activates the bilateral insula, MTG, bilateral MFG, bilateral IPL, mPFC, and precuneus. These brain regions have been previously linked to persuasion-induced behavior change (Falk et al., 2010). In addition, incongruent trials induced more activation than did congruent trials in the mPFC. This brain region have been associated with error-based learning and prediction error that leads to attitudinal and behavioral adjustments (Botvinick et al., 2001; Ridderinkhof et al., 2004; Preuschoff et al., 2008; Singer et al., 2009; Alexander and Brown, 2010; Cavanagh et al., 2010; Izuma et al., 2010; Ullsperger et al., 2010). Prediction error is defined as the difference between an expected and obtained outcome within reinforcement learning models (Schultz, 2006). Concerning social conformity, prediction error could be explained as a difference between individuals’ initial opinion and the belief of the group (Klucharev et al., 2009). In analyzing the conformity effect, we also found greater BOLD responses in the insula, bilateral MFG, and mPFC. Notably, the MFG, and mPFC also play a critical role in perceiving norm violations in the processing of social norms (Berthoz et al., 2002). As norm violation studies have suggested, the human brain may have developed specific mechanisms for detecting ongoing deviations from social norms (Montague and Lohrenz, 2007). Beer et al. (2003) found that patients with mPFC lesions were indifferent to social norms. Mason et al. (2009) reported that the mPFC was closely associated with normative social influence. The present fMRI results demonstrate that group opinions incongruent with a participant’s initial choice could trigger a neural process similar to norm violations, indicating deviations from group norms. Indeed, participants changed their performance and conformed to the group normative opinion when the deviation-related signal was active enough.

Finally, we found that the medial frontal gyrus was activated when people viewed the incongruent group opinion following an unfair offer which they rejected in the first decision phase. Previous neuroimaging studies have demonstrated that the MFG is involved in behavior change (Falk et al., 2010) and mentalizing functions, such as thinking about other people’s intentions, desires, and beliefs (Vogeley et al., 2004; Amodio and Frith, 2006). Previous literature has also reported that the capacity to understand other people’s behavioral intentions is crucial to regulating one’s social behavior to be according to group norms (Stallen et al., 2013). Considering the social implications of an unfair offer, one possible explanation is that individuals’ group identification increased when they believed that they have been treated unfairly (Branscombe et al., 1999, 2002). This strong group identification motivated individuals to take the viewpoints of the other group members, change their initial choices, and conform to group norms. The other possible explanation is that the incongruent-fair condition contains two social norms that conflict with each other (accept the fair offer versus conform to group opinions). This would make the conformity effect in the incongruent-fair condition easier to resist. Participants were more likely to conform to others’ behavior in the incongruent-unfair condition, because the incongruent-unfair condition presents the “conform to group opinion” norm more directly. Such reasoning is also consistent with the behavioral result that participants changed their initial choices when their initial choices were incongruent with the group opinion in unfair offer scenarios.

The present study complements previous research in assessing the neural basis of conformity and extends our understanding of economic decisions. Our behavioral results suggest that people change their choices due to a conflict with normative group opinions, especially when they were treated unfairly. In addition, our findings suggest that participants are more likely to conform to others’ behavior when they reject the unfair offer in the incongruent condition. The fMRI data indicate that the brain regions involved in norm violations and behavioral adjustment are activated when individuals encounter a divergent group opinion during a monetary game. The mechanisms underlying social conformity may be similar to those implicated in behavioral adjustments. 

## Conflict of Interest Statement

The authors declare that the research was conducted in the absence of any commercial or financial relationships that could be construed as a potential conflict of interest.

## References

[B1] Alexander W. H. Brown J. W. (2010). Computational models of performance monitoring and cognitive control. *Top. Cogn. Sci.* 2 658–677 10.1111/j.1756-8765.2010.01085.x 21359126 PMC3044326

[B2] Amodio D. M. Frith C. D. (2006). Meeting of minds: the medial frontal cortex and social cognition. *Nat. Rev. Neurosci.* 7 268–277 10.1038/nrn1884 16552413

[B3] Asch S. (1951). *Effects of Group Pressure Upon the Modification and Distortion of Judgments*. Pittsburgh: Carnegie Press

[B4] Beer J. S. Heerey E. H. Keltner D. Scabini D. Knight R. T. (2003). The regulatory function of self-conscious emotion: insights from patients with orbitofrontal damage. *J. Pers. Soc. Psychol.* 85 5946–5904 10.1037/0022-3514.85.4.594 14561114

[B5] Berns G. S. Capra C. M. Moore S. Noussair C. (2010). Neural mechanisms of the influence of popularity on adolescent ratings of music. *Neuroimage* 49 2687–2696 10.1016/j.neuroimage.2009.10.070 19879365 PMC2818406

[B6] Berns G. S. Chappelow J. Zink C. F. Pagnoni G. Martin-Skurski M. E. Richards J. (2005). Neurobiological correlates of social conformity and independence during mental rotation. *Biol. Psychiatry* 58 245–253 10.1016/j.biopsych.2005.04.012 15978553

[B7] Berns G. S. McClure S. M. Pagnoni G. Montague P. R. (2001). Predictability modulates human brain response to reward. *J. Neurosci.* 21 2793–2798 11306631 10.1523/JNEUROSCI.21-08-02793.2001 PMC6762527

[B8] Berthoz S. Armony J. L. Blair R. J. J. Dolan R. J. (2002). An fMRI study of intentional and unintentional (embarrassing) violations of social norms. *Brain* 125 1696–1708 10.1093/brain/awf190 12135962

[B9] Bolton G. E. Zwic R. (1995). Anonymity versus punishment in ultimatum bargaining. *Games Econ. Behav.* 10 95–121 10.1006/game.1995.1026

[B10] Bornstein G. Yaniv I. (1998). Individual and group behavior in the ultimatum game: are groups more “rational” players. *Exp. Econ.* 1 101–108 10.1007/BF01426217

[B11] Botvinick M. M. Braver T. S. Barch D. M. Carter C. S. Cohen J. D. (2001). Conflict monitoring and cognitive control. *Psychol. Rev.* 108 624–652 10.1037/0033-295X.108.3.624 11488380

[B12] Branscombe N. R. Schmitt M. T. Harvey R. D. (1999). Perceiving pervasive discrimination among African Americans: implications for group identification and well-being. *J. Pers. Soc. Psychol.* 77 135–149 10.1037/0022-3514.77.1.135

[B13] Branscombe N. R. Spears R. Ellemers N. Doosje B. (2002). Intragroup and intergroup evaluation effects on group behavior. *Pers. Soc. Psychol. Bull.* 28 744–753 10.1177/0146167202289004

[B14] Camerer C. F. (2003). Behavioural studies of strategic thinking in games. *Trends Cogn. Sci.* 7 225–231 10.1016/S1364-6613(03)00094-9 12757825

[B15] Campbell-Meiklejohn D. Bach D. Roepstorff A. (2010). How the opinion of others affects our valuation of objects. *Curr. Biol.* 20 1165–1170 10.1016/j.cub.2010.04.055 20619815 PMC2908235

[B16] Cavanagh J. F. Frank M. J. Klein T. J. Allen J. J. (2010). Frontal theta links prediction errors to behavioral adaptation in reinforcement learning. *Neuroimage* 49 3198–3209 10.1016/j.neuroimage.2009.11.080 19969093 PMC2818688

[B17] Cialdini R. B. Goldstein N. J. (2004). Social influence: compliance and conformity. *Annu. Rev. Psychol.* 55 591–621 10.1146/annurev.psych.55.090902.142015 14744228

[B18] Falk E. B. Berkman E. T. Mann T. Harrison B. Lieberman M. D. (2010). Predicting persuasion-induced behavior change from the brain. *J. Neurosci.* 30 8421–8424 10.1523/JNEUROSCI.0063-10.2010 20573889 PMC3027351

[B19] Güth R. Schmittberger B. Schwarze B. (1982). An experimental analysis of ultimatum bargaining. *J. Econ. Behav. Organ.* 3 367–388 10.1016/0167-2681(82)90011-7

[B20] Haun D van Leeuwen E. J. (2012). Majority influence in children and other animals. *Dev. Cogn. Neurosci.* 3 61–71 10.1016/j.dcn.2012.09.003 23245221 PMC6987688

[B21] Holroyd C. B. Coles M. G. (2002). The neural basis of human error processing: reinforcement learning, dopamine, and the error-related negativity. *Psychol. Rev.* 109 679–709 10.1037/0033-295X.109.4.679 12374324

[B22] Izuma K. Matsumoto M. Murayama K. Samejima K. Sadato N. Matsumoto K. (2010). Neural correlates of cognitive dissonance and choice-induced preference change. *Proc. Natl. Acad. Sci. U.S.A.* 107 22014–22019 10.1073/pnas.1011879108 21135218 PMC3009797

[B23] Klucharev V. Hytönen K. Rijpkema M. Smidts A. Fernández G. (2009). Reinforcement learning signal predicts social conformity. *Neuron* 61 140–151 10.1016/j.neuron.2008.11.027 19146819

[B24] Klucharev V. Munneke M. A. Smidts A. Fernández G. (2011). Downregulation of the posterior medial frontal cortex prevents social conformity. *J. Neurosci.* 31 11934–11940 10.1523/JNEUROSCI.1869-11.2011 21849554 PMC6623179

[B25] Mason M. F. Dyer R. Norton M. I. (2009). Neural mechanisms of social influence. *Organ. Behav. Hum. Decis. Process.* 110 152–159 10.1016/j.obhdp.2009.04.001

[B26] Montague P. R. Lohrenz T. (2007). To detect and correct: norm violations and their enforcement. *Neuron* 56 14–18 10.1016/j.neuron.2007.09.020 17920011

[B27] Morgan T. J. H. Laland K. (2012). The biological bases of conformity. *Front. Neurosci.* 6:87 10.3389/fnins.2012.00087 PMC3375089 22712006

[B28] Nowak M. A. Page K. M. Sigmund K. (2000). Fairness versus reason in the ultimatum game. *Science* 289 1773–1775 10.1126/science.289.5485.1773 10976075

[B29] Oosterbeek H. Sloof R Van de Kuilen G. (2003). Cultural differences in ultimatum game experiments: evidence from a meta-analysis. *Exp. Econ.* 7 171–188 10.1023/B:EXEC.0000026978.14316.74

[B30] Preuschoff K. Quartz S. R. Bossaerts P. (2008). Human insula activation reflects risk prediction errors as well as risk. *J. Neurosci.* 28 2745–2752 10.1523/JNEUROSCI.4286-07.2008 18337404 PMC6670675

[B31] Ridderinkhof K. R. Ullsperger M. Crone E. A. Nieuwenhuis S. (2004). The role of the medial frontal cortex in cognitive control. *Science* 306 443–447 10.1126/science.1100301 15486290

[B32] Sanfey A. G. (2007). Social decision-making: insights from game theory and neuroscience. *Science* 318 598–602 10.1126/science.1142996 17962552

[B33] Schultz W. (2006). Behavioral theories and the neurophysiology of reward. *Annu. Rev. Psychol.* 57 87–115 10.1146/annurev.psych.56.091103.070229 16318590

[B34] Singer T. Critchley H. D. Preuschoff K. (2009). A common role of insula in feelings, empathy and uncertainty. *Trends Cogn. Sci.* 13 334–340 10.1016/j.tics.2009.05.001 19643659

[B35] Song X. W. Dong Z. Y. Long X. Y. Li S. F. Zuo X. N. Zhu C. Z. (2011). REST: a toolkit for resting-state functional magnetic resonance imaging data processing. *PLoS ONE* 6:e25031 10.1371/journal.pone.0025031 PMC3176805 21949842

[B36] Stallen M. Smidts A. Sanfey A. G. (2013). Peer influence: neural mechanisms underlying in-group conformity. *Front. Hum. Neurosci.* 7:50 10.3389/fnhum.2013.00050 PMC3591747 23482688

[B37] Turner J. C. (1991). *Social Influence*. London: Open University Press

[B38] Ullsperger M. Harsay H. A. Wessel J. R. Ridderinkhof K. R. (2010). Conscious perception of errors and its relation to the anterior insula. *Brain Struct. Funct.* 214 629–643 10.1007/s00429-010-0261-1 20512371 PMC2886909

[B39] Van Essen D. C. (2005). A Population-Average, Landmark- and Surface-based (PALS) atlas of human cerebral cortex. *Neuroimage* 28 635–662 10.1016/j.neuroimage.2005.06.058 16172003

[B40] Vogeley K. May M. Ritzl A. Falkai P. Zilles K. Fink G. R. (2004). Neural correlates of first-person perspective as one constituent of human self-consciousness. *J. Cogn. Neurosci.* 16 817–827 10.1162/089892904970799 15200709

[B41] Zaki J. Schirmer J. Mitchell J. P. (2011). Social influence modulates the neural computation of value. *Psychol. Sci.* 22 894–900 10.1177/0956797611411057 21653908

